# 1-[(5-Bromo­pent­yloxy)meth­yl]pyrene

**DOI:** 10.1107/S1600536811018253

**Published:** 2011-05-20

**Authors:** Xixi Bian, Tongjiang Cai, Jin Zhou, Qin Huang, Xunwen Xiao

**Affiliations:** a Ningbo University of Technology, Ningbo 315016, People’s Republic of China

## Abstract

In the title compound, C_22_H_21_BrO, other than the Br atom, the non-H atoms are approximately co-planar [maxium deviation = 0.178 (2) Å] and the alk­oxy chain shows an all-*anti* conformation. A weak inter­molecular C—H⋯Br hydrogen bond contributes to the stabilization of the crystal structure.

## Related literature

For the synthesis of pyrene derivatives, see Filby & Steed (2006 ▶). For the use of pyrenes as fluorescence sensors, see: Bell & Hext (2004 ▶). For related structures, see: Fun *et al.* (2009 ▶); Gruber *et al.* (2010 ▶); Xiao *et al.* (2005 ▶).
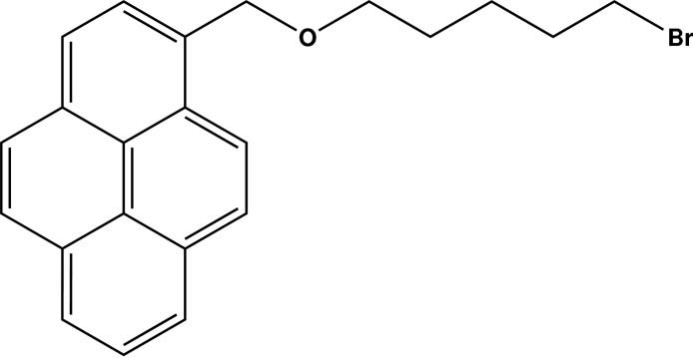


## Experimental

### 

#### Crystal data

C_22_H_21_BrO
*M* *_r_* = 381.30
Triclinic, 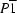

*a* = 7.417 (2) Å
*b* = 7.4817 (16) Å
*c* = 17.545 (5) Å
α = 79.924 (19)°
β = 88.90 (2)°
γ = 64.295 (12)°
*V* = 861.9 (4) Å^3^
*Z* = 2
Mo *K*α radiation
μ = 2.39 mm^−1^
*T* = 223 K
0.45 × 0.40 × 0.20 mm

#### Data collection

Rigaku Saturn diffractometer
Absorption correction: multi-scan (REQAB; Jacobson, 1998 ▶) *T*_min_ = 0.373, *T*_max_ = 0.616
7097 measured reflections
3085 independent reflections
2399 reflections with *I* > 2σ(*I*)
*R*_int_ = 0.038

#### Refinement

*R*[*F* ^2^ > 2σ(*F* ^2^)] = 0.028
*wR*(*F* ^2^) = 0.047
*S* = 0.88
3085 reflections
218 parameters
H-atom parameters constrained
Δρ_max_ = 0.31 e Å^−3^
Δρ_min_ = −0.36 e Å^−3^

### 

Data collection: *CrystalClear* (Rigaku, 2005 ▶); cell refinement: *CrystalClear*; data reduction: *CrystalStructure* (Rigaku, 2005 ▶); program(s) used to solve structure: *SHELXS97* (Sheldrick, 2008 ▶); program(s) used to refine structure: *SHELXL97* (Sheldrick, 2008 ▶); molecular graphics: *ORTEP-3* (Farrugia, 1997 ▶); software used to prepare material for publication: *SHELXTL* (Sheldrick, 2008 ▶).

## Supplementary Material

Crystal structure: contains datablocks I, global. DOI: 10.1107/S1600536811018253/ng5165sup1.cif

Structure factors: contains datablocks I. DOI: 10.1107/S1600536811018253/ng5165Isup2.hkl

Supplementary material file. DOI: 10.1107/S1600536811018253/ng5165Isup3.cml

Additional supplementary materials:  crystallographic information; 3D view; checkCIF report

## Figures and Tables

**Table 1:** Hydrogen-bond geometry (Å, °)

*D*—H⋯*A*	*D*—H	H⋯*A*	*D*⋯*A*	*D*—H⋯*A*
C6—H6⋯Br1^i^	0.94	3.02	3.869 (2)	151

Symmetry code: (i) 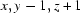.

**Fig. 1.**
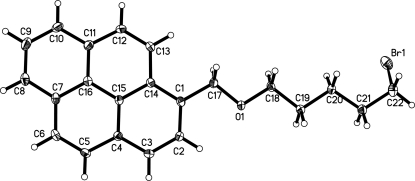
The molecular structure of the title compound.Displacement ellipsoids are drawn at the 30% probability level.

**Fig. 2.**
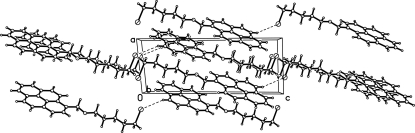
The crystal packing diagram view along the crystallographic 〈i〉b〈/i〉 axis. Dashed lines indicate the hydrogen bonding.

## supplementary crystallographic information

### Comment

As a fluorogenic unit, pyrene is one of the most useful fluorescene probe because of its relatively efficient excimer formation and emission. In this respect, the title compound was prepared as part of our research on the solid state structure of fluorogenic tetrathiafulvalene with possible molecular switch (Xiao *et al.*., 2005).

The bond lengths and bond angles of the title compound are found to have norml values (Fun *et al.*., 2009 Gruber *et al.*., 2010). Expect the Br atom and H atoms, the molecule is essentially planar with the maximum deviation from planarity being 0.1781 (21) Å. In the substitute alkoxy chain, expect the Br atom, it shows the typical all-anti conformation (Fig.1).

The crystal packing is stabilized by C—H···Br intermolecular hydrogen bonding (table.1) (Fig.2).

### Experimental

The title compound was synthesiaed according to a literature procedure (Xiao *et al.*., 2005). Slow evaporation of a solution in THF gave single crystals suitable for *X*–ray analysis.

### Refinement

The H atoms were positioned geometrically and allowed to ride on their parent atoms, with C—H = 0.95–0.99Å and *U*_iso_ = 1.2–1.5 *U*_eq_(parent atom).

### Crystal data

**Table:**

C_22_H_21_BrO	*Z* = 2
*M**_r_* = 381.30	*F*(000) = 392
Triclinic, *P*1	*D*_x_ = 1.469 Mg m^−^^3^
Hall symbol: -P 1	Mo *K*α radiation, λ = 0.71075 Å
*a* = 7.417 (2) Å	Cell parameters from 4242 reflections
*b* = 7.4817 (16) Å	θ = 3.1–27.5°
*c* = 17.545 (5) Å	µ = 2.39 mm^−^^1^
α = 79.924 (19)°	*T* = 223 K
β = 88.90 (2)°	Block, colorless
γ = 64.295 (12)°	0.45 × 0.40 × 0.20 mm
*V* = 861.9 (4) Å^3^	

### Data collection

**Table:**

Rigaku Saturn diffractometer	3085 independent reflections
Radiation source: fine-focus sealed tube	2399 reflections with *I* > 2σ(*I*)
graphite	*R*_int_ = 0.038
Detector resolution: 14.63 pixels mm^-1^	θ_max_ = 25.5°, θ_min_ = 3.1°
ω scans	*h* = −8→8
Absorption correction: multi-scan (REQAB; Jacobson, 1998)	*k* = −9→9
*T*_min_ = 0.373, *T*_max_ = 0.616	*l* = −21→21
7097 measured reflections	

### Refinement

**Table:**

Refinement on *F*^2^	Primary atom site location: structure-invariant direct methods
Least-squares matrix: full	Secondary atom site location: difference Fourier map
*R*[*F*^2^ > 2σ(*F*^2^)] = 0.028	Hydrogen site location: inferred from neighbouring sites
*wR*(*F*^2^) = 0.047	H-atom parameters constrained
*S* = 0.88	*w* = 1/[σ^2^(*F*_o_^2^) + (0.0129*P*)^2^] where *P* = (*F*_o_^2^ + 2*F*_c_^2^)/3
3085 reflections	(Δ/σ)_max_ < 0.001
218 parameters	Δρ_max_ = 0.31 e Å^−^^3^
0 restraints	Δρ_min_ = −0.36 e Å^−^^3^

### Special details

**Table:**

Geometry. All e.s.d.'s (except the e.s.d. in the dihedral angle between two l.s. planes) are estimated using the full covariance matrix. The cell e.s.d.'s are taken into account individually in the estimation of e.s.d.'s in distances, angles and torsion angles; correlations between e.s.d.'s in cell parameters are only used when they are defined by crystal symmetry. An approximate (isotropic) treatment of cell e.s.d.'s is used for estimating e.s.d.'s involving l.s. planes.
Refinement. Refinement of *F*^2^ against ALL reflections. The weighted *R*-factor *wR* and goodness of fit *S* are based on *F*^2^, conventional *R*-factors *R* are based on *F*, with *F* set to zero for negative *F*^2^. The threshold expression of *F*^2^ > σ(*F*^2^) is used only for calculating *R*-factors(gt) *etc*. and is not relevant to the choice of reflections for refinement. *R*-factors based on *F*^2^ are statistically about twice as large as those based on *F*, and *R*- factors based on ALL data will be even larger.

### Fractional atomic coordinates and isotropic or equivalent isotropic displacement parameters (Å^2^)

**Table:**

	*x*	*y*	*z*	*U*_iso_*/*U*_eq_	
Br1	0.30010 (4)	0.85258 (4)	0.015557 (11)	0.04908 (9)	
O1	0.35680 (18)	0.46204 (17)	0.40464 (6)	0.0263 (3)	
C1	0.2422 (3)	0.4448 (3)	0.53439 (9)	0.0217 (4)	
C2	0.3303 (3)	0.2367 (3)	0.54290 (9)	0.0246 (4)	
H2	0.3941	0.1767	0.5010	0.030*	
C3	0.3272 (3)	0.1144 (3)	0.61153 (9)	0.0272 (4)	
H3	0.3891	−0.0266	0.6156	0.033*	
C4	0.2324 (3)	0.1992 (3)	0.67517 (9)	0.0222 (4)	
C5	0.2242 (3)	0.0795 (3)	0.74734 (9)	0.0278 (4)	
H5	0.2844	−0.0619	0.7528	0.033*	
C6	0.1319 (3)	0.1649 (3)	0.80776 (9)	0.0285 (4)	
H6	0.1286	0.0818	0.8541	0.034*	
C7	0.0391 (3)	0.3794 (3)	0.80237 (9)	0.0250 (4)	
C8	−0.0539 (3)	0.4721 (3)	0.86452 (10)	0.0308 (5)	
H8	−0.0562	0.3920	0.9119	0.037*	
C9	−0.1421 (3)	0.6798 (3)	0.85704 (10)	0.0368 (5)	
H9	−0.2031	0.7393	0.8994	0.044*	
C10	−0.1415 (3)	0.8005 (3)	0.78816 (10)	0.0344 (5)	
H10	−0.2032	0.9416	0.7840	0.041*	
C11	−0.0503 (3)	0.7163 (3)	0.72412 (9)	0.0266 (4)	
C12	−0.0449 (3)	0.8358 (3)	0.65197 (10)	0.0300 (4)	
H12	−0.1070	0.9772	0.6464	0.036*	
C13	0.0471 (3)	0.7513 (3)	0.59160 (10)	0.0274 (4)	
H13	0.0481	0.8352	0.5451	0.033*	
C14	0.1431 (3)	0.5368 (3)	0.59663 (9)	0.0209 (4)	
C15	0.1394 (2)	0.4125 (3)	0.66725 (9)	0.0203 (4)	
C16	0.0418 (3)	0.5033 (3)	0.73151 (9)	0.0218 (4)	
C17	0.2486 (3)	0.5781 (3)	0.46037 (9)	0.0248 (4)	
H17A	0.1114	0.6679	0.4390	0.030*	
H17B	0.3122	0.6620	0.4716	0.030*	
C18	0.3537 (3)	0.5952 (3)	0.33503 (9)	0.0240 (4)	
H18A	0.4167	0.6799	0.3461	0.029*	
H18B	0.2145	0.6839	0.3158	0.029*	
C19	0.4640 (3)	0.4774 (3)	0.27364 (9)	0.0238 (4)	
H19A	0.4014	0.3925	0.2625	0.029*	
H19B	0.6035	0.3893	0.2925	0.029*	
C20	0.4582 (3)	0.6222 (3)	0.19985 (9)	0.0254 (4)	
H20A	0.3180	0.7134	0.1830	0.030*	
H20B	0.5232	0.7045	0.2117	0.030*	
C21	0.5605 (3)	0.5177 (3)	0.13337 (9)	0.0289 (4)	
H21A	0.6977	0.4188	0.1515	0.035*	
H21B	0.4890	0.4438	0.1188	0.035*	
C22	0.5683 (3)	0.6598 (3)	0.06263 (10)	0.0380 (5)	
H22A	0.6453	0.5811	0.0242	0.046*	
H22B	0.6387	0.7348	0.0770	0.046*	

### Atomic displacement parameters (Å^2^)

**Table:**

	*U*^11^	*U*^22^	*U*^33^	*U*^12^	*U*^13^	*U*^23^
Br1	0.06041 (17)	0.05227 (16)	0.02991 (11)	−0.02606 (13)	−0.00843 (10)	0.00909 (9)
O1	0.0320 (8)	0.0235 (7)	0.0188 (6)	−0.0089 (6)	0.0073 (6)	−0.0022 (5)
C1	0.0203 (10)	0.0251 (11)	0.0197 (8)	−0.0110 (9)	−0.0006 (7)	−0.0007 (8)
C2	0.0273 (11)	0.0246 (11)	0.0209 (8)	−0.0098 (9)	0.0045 (8)	−0.0058 (8)
C3	0.0318 (12)	0.0187 (11)	0.0280 (9)	−0.0085 (9)	0.0022 (8)	−0.0038 (8)
C4	0.0226 (10)	0.0226 (11)	0.0210 (8)	−0.0101 (9)	−0.0007 (8)	−0.0027 (8)
C5	0.0329 (12)	0.0193 (11)	0.0274 (9)	−0.0099 (10)	−0.0009 (9)	0.0016 (8)
C6	0.0323 (12)	0.0316 (12)	0.0214 (9)	−0.0170 (10)	0.0001 (8)	0.0037 (8)
C7	0.0243 (11)	0.0351 (12)	0.0199 (8)	−0.0173 (10)	0.0025 (8)	−0.0042 (8)
C8	0.0331 (12)	0.0414 (13)	0.0229 (9)	−0.0217 (11)	0.0066 (9)	−0.0039 (9)
C9	0.0422 (13)	0.0464 (14)	0.0310 (10)	−0.0243 (12)	0.0161 (10)	−0.0184 (10)
C10	0.0384 (13)	0.0284 (12)	0.0384 (11)	−0.0135 (10)	0.0135 (10)	−0.0154 (9)
C11	0.0274 (11)	0.0275 (12)	0.0274 (9)	−0.0133 (10)	0.0052 (8)	−0.0084 (8)
C12	0.0350 (12)	0.0179 (11)	0.0333 (10)	−0.0076 (10)	0.0082 (9)	−0.0066 (8)
C13	0.0332 (11)	0.0240 (11)	0.0223 (9)	−0.0119 (10)	0.0025 (8)	0.0005 (8)
C14	0.0202 (10)	0.0225 (11)	0.0199 (8)	−0.0100 (9)	−0.0005 (7)	−0.0024 (8)
C15	0.0192 (10)	0.0236 (11)	0.0186 (8)	−0.0101 (9)	0.0003 (7)	−0.0026 (7)
C16	0.0199 (10)	0.0257 (11)	0.0214 (8)	−0.0118 (9)	0.0005 (7)	−0.0037 (8)
C17	0.0255 (11)	0.0267 (11)	0.0206 (8)	−0.0099 (9)	0.0036 (8)	−0.0044 (8)
C18	0.0272 (11)	0.0251 (11)	0.0179 (8)	−0.0114 (9)	0.0013 (8)	0.0008 (8)
C19	0.0255 (11)	0.0236 (11)	0.0215 (8)	−0.0106 (9)	0.0020 (8)	−0.0022 (8)
C20	0.0287 (11)	0.0274 (11)	0.0200 (8)	−0.0121 (9)	0.0049 (8)	−0.0054 (8)
C21	0.0320 (11)	0.0321 (12)	0.0216 (8)	−0.0139 (10)	0.0039 (8)	−0.0033 (8)
C22	0.0406 (13)	0.0491 (14)	0.0252 (9)	−0.0213 (11)	0.0063 (9)	−0.0050 (9)

### Geometric parameters (Å, °)

**Table:**

Br1—C22	1.969 (2)	C11—C16	1.418 (2)
O1—C17	1.4200 (19)	C11—C12	1.427 (2)
O1—C18	1.4283 (19)	C12—C13	1.349 (2)
C1—C2	1.383 (2)	C12—H12	0.9400
C1—C14	1.414 (2)	C13—C14	1.433 (2)
C1—C17	1.506 (2)	C13—H13	0.9400
C2—C3	1.386 (2)	C14—C15	1.421 (2)
C2—H2	0.9400	C15—C16	1.437 (2)
C3—C4	1.405 (2)	C17—H17A	0.9800
C3—H3	0.9400	C17—H17B	0.9800
C4—C15	1.419 (2)	C18—C19	1.510 (2)
C4—C5	1.434 (2)	C18—H18A	0.9800
C5—C6	1.353 (2)	C18—H18B	0.9800
C5—H5	0.9400	C19—C20	1.524 (2)
C6—C7	1.432 (3)	C19—H19A	0.9800
C6—H6	0.9400	C19—H19B	0.9800
C7—C8	1.402 (2)	C20—C21	1.523 (2)
C7—C16	1.420 (2)	C20—H20A	0.9800
C8—C9	1.382 (3)	C20—H20B	0.9800
C8—H8	0.9400	C21—C22	1.504 (2)
C9—C10	1.378 (3)	C21—H21A	0.9800
C9—H9	0.9400	C21—H21B	0.9800
C10—C11	1.402 (2)	C22—H22A	0.9800
C10—H10	0.9400	C22—H22B	0.9800
			
C17—O1—C18	109.08 (13)	C4—C15—C14	120.57 (15)
C2—C1—C14	119.58 (16)	C4—C15—C16	119.62 (15)
C2—C1—C17	121.89 (15)	C14—C15—C16	119.80 (16)
C14—C1—C17	118.53 (16)	C11—C16—C7	120.18 (15)
C1—C2—C3	121.76 (16)	C11—C16—C15	119.89 (16)
C1—C2—H2	119.1	C7—C16—C15	119.94 (16)
C3—C2—H2	119.1	O1—C17—C1	111.32 (14)
C2—C3—C4	120.58 (17)	O1—C17—H17A	109.4
C2—C3—H3	119.7	C1—C17—H17A	109.4
C4—C3—H3	119.7	O1—C17—H17B	109.4
C3—C4—C15	118.45 (15)	C1—C17—H17B	109.4
C3—C4—C5	122.80 (16)	H17A—C17—H17B	108.0
C15—C4—C5	118.75 (15)	O1—C18—C19	110.73 (14)
C6—C5—C4	121.65 (17)	O1—C18—H18A	109.5
C6—C5—H5	119.2	C19—C18—H18A	109.5
C4—C5—H5	119.2	O1—C18—H18B	109.5
C5—C6—C7	121.20 (16)	C19—C18—H18B	109.5
C5—C6—H6	119.4	H18A—C18—H18B	108.1
C7—C6—H6	119.4	C18—C19—C20	109.88 (15)
C8—C7—C16	118.66 (17)	C18—C19—H19A	109.7
C8—C7—C6	122.51 (16)	C20—C19—H19A	109.7
C16—C7—C6	118.83 (15)	C18—C19—H19B	109.7
C9—C8—C7	120.82 (17)	C20—C19—H19B	109.7
C9—C8—H8	119.6	H19A—C19—H19B	108.2
C7—C8—H8	119.6	C21—C20—C19	113.91 (15)
C10—C9—C8	120.69 (16)	C21—C20—H20A	108.8
C10—C9—H9	119.7	C19—C20—H20A	108.8
C8—C9—H9	119.7	C21—C20—H20B	108.8
C9—C10—C11	121.02 (18)	C19—C20—H20B	108.8
C9—C10—H10	119.5	H20A—C20—H20B	107.7
C11—C10—H10	119.5	C22—C21—C20	113.79 (15)
C10—C11—C16	118.63 (16)	C22—C21—H21A	108.8
C10—C11—C12	122.79 (17)	C20—C21—H21A	108.8
C16—C11—C12	118.58 (15)	C22—C21—H21B	108.8
C13—C12—C11	121.76 (17)	C20—C21—H21B	108.8
C13—C12—H12	119.1	H21A—C21—H21B	107.7
C11—C12—H12	119.1	C21—C22—Br1	112.67 (13)
C12—C13—C14	121.56 (16)	C21—C22—H22A	109.1
C12—C13—H13	119.2	Br1—C22—H22A	109.1
C14—C13—H13	119.2	C21—C22—H22B	109.1
C1—C14—C15	119.04 (16)	Br1—C22—H22B	109.1
C1—C14—C13	122.55 (16)	H22A—C22—H22B	107.8
C15—C14—C13	118.41 (15)		
			
C14—C1—C2—C3	−1.0 (3)	C3—C4—C15—C16	−179.80 (15)
C17—C1—C2—C3	178.85 (15)	C5—C4—C15—C16	0.8 (2)
C1—C2—C3—C4	0.3 (3)	C1—C14—C15—C4	−0.4 (2)
C2—C3—C4—C15	0.3 (2)	C13—C14—C15—C4	−179.97 (15)
C2—C3—C4—C5	179.71 (16)	C1—C14—C15—C16	179.12 (14)
C3—C4—C5—C6	−179.99 (16)	C13—C14—C15—C16	−0.5 (2)
C15—C4—C5—C6	−0.6 (2)	C10—C11—C16—C7	0.6 (3)
C4—C5—C6—C7	−0.4 (3)	C12—C11—C16—C7	−179.87 (15)
C5—C6—C7—C8	−178.58 (16)	C10—C11—C16—C15	−178.99 (15)
C5—C6—C7—C16	1.2 (3)	C12—C11—C16—C15	0.5 (2)
C16—C7—C8—C9	0.3 (3)	C8—C7—C16—C11	−0.8 (2)
C6—C7—C8—C9	−179.91 (17)	C6—C7—C16—C11	179.42 (16)
C7—C8—C9—C10	0.3 (3)	C8—C7—C16—C15	178.81 (15)
C8—C9—C10—C11	−0.5 (3)	C6—C7—C16—C15	−0.9 (2)
C9—C10—C11—C16	0.0 (3)	C4—C15—C16—C11	179.61 (15)
C9—C10—C11—C12	−179.44 (17)	C14—C15—C16—C11	0.1 (2)
C10—C11—C12—C13	178.72 (17)	C4—C15—C16—C7	0.0 (2)
C16—C11—C12—C13	−0.7 (3)	C14—C15—C16—C7	−179.54 (14)
C11—C12—C13—C14	0.4 (3)	C18—O1—C17—C1	178.43 (13)
C2—C1—C14—C15	1.0 (2)	C2—C1—C17—O1	−1.1 (2)
C17—C1—C14—C15	−178.83 (14)	C14—C1—C17—O1	178.76 (13)
C2—C1—C14—C13	−179.42 (16)	C17—O1—C18—C19	−178.88 (13)
C17—C1—C14—C13	0.7 (2)	O1—C18—C19—C20	179.77 (13)
C12—C13—C14—C1	−179.34 (16)	C18—C19—C20—C21	−178.27 (14)
C12—C13—C14—C15	0.2 (3)	C19—C20—C21—C22	−175.62 (14)
C3—C4—C15—C14	−0.3 (2)	C20—C21—C22—Br1	−63.25 (17)
C5—C4—C15—C14	−179.68 (14)		

### Hydrogen-bond geometry (Å, °)

**Table:**

*D*—H···*A*	*D*—H	H···*A*	*D*···*A*	*D*—H···*A*
C6—H6···Br1^i^	0.94	3.02	3.869 (2)	151

Symmetry codes: (i) *x*, *y*−1, *z*+1.
